# The role of the Alzheimer's Disease Neuroimaging Initiative in establishing the Dominantly Inherited Alzheimer Network

**DOI:** 10.1002/alz.14106

**Published:** 2024-07-03

**Authors:** John C. Morris, Virginia D. Buckles

**Affiliations:** ^1^ Department of Neurology Washington University School of Medicine St. Louis Missouri USA; ^2^ Knight Alzheimer Disease Research Center Washington University School of Medicine Saint Louis Missouri USA

**Keywords:** Alzheimer disease biomarkers, autosomal dominant Alzheimer's disease, late onset Alzheimer's disease

## Abstract

The Dominantly Inherited Alzheimer Network (DIAN) initially was funded by the National Institute on Aging (NIA) in 2008 and thus was able to adopt and incorporate the protocols developed by the Alzheimer's Disease Neuroimaging Initiative (ADNI) that had been established by the NIA in 2004. The use of ADNI protocols for DIAN neuroimaging studies and assays of biological fluids for Alzheimer disease (AD) biomarkers permitted examination of the hypothesis that autosomal dominant AD (ADAD), studied by DIAN, and “sporadic” late‐onset AD (LOAD), studied by ADNI, shared the same pathobiological construct. In a collaborative effort, the longitudinal DIAN and ADNI databases were compared and the findings supported the conclusion that ADAD and LOAD share a similar pathophysiology. The importance of the DIAN study thus is amplified by its relevance to LOAD, as characterized by the “parent” ADNI program.

## 1 INTRODUCTION

As the experimental therapeutic landscape for Alzheimer disease (AD) expanded in the early 2000s to incorporate putative disease‐modifying therapies,^1^ Neil Buckholtz, PhD, Chief of the Dementias of Aging Branch of the Division of Neuroscience at the National Institute of Aging (NIA), envisioned a major effort to collect data and samples to establish a brain imaging, biomarker, and clinical database that would permit the identification and validation of markers to facilitate interventional studies of these new potential therapies for AD. That vision was realized in 2004 when the NIA funded an application to establish the Alzheimer's Disease Neuroimaging Initiative (ADNI; U19 AG024904; MW Weiner, PI). The major goals of this longitudinal multi‐site observational study were and are to: (1) develop the optimum methods for obtaining, processing, and distributing images and biomarkers in conjunction with clinical and cognitive data across multiple sites; (2) to validate the imaging and biomarker data; and (3) to provide rapid public access to all data and samples. Some 60 centers in North America agreed to serve as ADNI performance sites and the study was launched with the enrollment of its first participant at the ADNI site at Washington University in St Louis in August 2005.

During this same period, the Knight Alzheimer Disease Research Center (ADRC) at Washington University increasingly was focused on identifying and characterizing the asymptomatic preclinical stage of AD, when AD pathology is present in the brain but has yet to cause sufficient synaptic and neuronal injury to permit AD symptoms to appear.^2^, ^3^, ^4^ To that end, the Knight ADRC began a pilot study in 2002 to enroll and follow a middle‐aged (45 years and older) cohort of cognitively normal persons who were at elevated risk of developing preclinical and symptomatic AD due to a parental history of AD dementia developing before 80 years.

The pilot study indicated that initial biomarker evidence of AD could be detected between ages 45 and 50 and served to support a successful application in 2005 to establish the program project, “Antecedent Biomarkers of Alzheimer Disease: The Adult Children Study” (ACS; P01 AG026276; JC Morris, PI). The ACS participants agreed to undergo, at baseline and every 3 years thereafter, biomarker procedures that included structural brain imaging, positron emission tomography (PET) with the amyloid radiotracer, Pittsburgh Compound‐B (PIB), and lumbar puncture to obtain cerebrospinal fluid (CSF) for biomarker assays. A competitive supplement, led by Randall J. Bateman, to the ACS program project grant was funded in 2006 to establish a special group of adult children with parents whose AD dementia was caused by rare deterministic mutations in the *amyloid precursor protein*, *presenilin 1*, or *presenilin 2* genes. Also in 2006, Dr. Buckholtz attended an NIA conference, held 100 years after the 37th Assembly of Southwest German Psychiatrists in Tϋbingen where Alois Alzheimer presented the first known case of the illness that now bears his name, to set the future research agenda on AD and related dementias. One recommendation that came from the conference was to study dominantly inherited AD. In response, Dr. Buckholtz wrote a Request for Applications, issued on 7/17/2007 (RFA‐AG‐08‐002), to create an international consortium to study individuals from families with a known causative mutation for AD. This international network of scientific investigators would “identify, recruit, evaluate, and follow individuals” from these rare families with dominantly inherited AD as they offered the certainty of diagnosis (the mutations are virtually 100% penetrant), the opportunity to assess mutation carriers in the preclinical stage of AD, and the relative absence of age‐associated comorbidities (the mean age of symptomatic onset of AD in these families is ≅47 years). It was assumed that information from this rare form of dominantly inherited AD “would also be relevant to late‐onset disease.” The RFA encouraged the consideration of ADNI as an exemplar of a multi‐site observational study and further specified that the “protocols used in this study should be consistent with those developed by [ADNI] for data and sample collection, storage, and analysis.” Finally, Dr. Buckholtz encouraged Dr. Morris from Washington University to assemble an international team and submit an application in response to the RFA (Neil Buckholtz, personal communication).

Morris recruited nine centers in the United States, United Kingdom, and Australia to join Washington University to form the Dominantly Inherited Alzheimer Network (DIAN). The resulting application was favorably reviewed and NIA funding began on September 18, 2008, for an initial 6‐year period (U01 AG032438; JC Morris, PI). The grant addressed three major hypotheses: (1) preclinical AD in mutation carriers can be detected by biomarker differences in biological fluids and neuroimaging measures when compared with their noncarrier siblings; (2) the sequence of preclinical changes is initiated by alterations in cerebral levels of amyloid‐beta‐42 (Aβ_42_), as detected by reduced Aβ42 levels in the CSF and by cerebral deposition of Aβ_42_ in the form of plaques (amyloid PET imaging); and (3) the phenotype of autosomal dominant AD (ADAD) is similar to that of “sporadic” late‐onset AD (LOAD). As the RFA instructed, DIAN adopted the ADNI protocols for the imaging and fluid biomarker studies.

DIAN recruited and enrolled biological adult children, both mutation carriers and noncarriers, of a parent with a known causative mutation for AD.^5^ All participants were evaluated at baseline and longitudinally thereafter in a uniform manner with protocols that included:

1. The clinical and cognitive assessments of the Uniform Data Set^6^, ^7^ used by all NIA‐funded Alzheimer Disease Research Centers in the United States.
2. The ADNI structural, functional, and amyloid imaging protocols.
3. The ADNI protocols for the collection of biological fluids (CSF; blood) for DNA analysis and assays of AD biomarkers.
4. Neuropathological assessment of cerebral tissue of participants who came to autopsy.

DIAN established an integrated database to facilitate analyses within, between, and among the various data domains and to disseminate the data to qualified investigators, while maintaining the confidentiality of all data, including mutation status. Genetic counseling was provided to DIAN participants who wished to learn their mutation status, which was determined by genetic testing in Clinical Laboratory Improvement Amendment‐approved laboratories.

Selection of the original DIAN sites was restricted to English‐speaking centers to avoid the added complications of translating protocols and work flows in the initial years of the consortium. Once established, DIAN expanded to include additional sites in Europe (including Germany and Spain), South America (originally Argentina and recently expanded to multiple Latin American sites), and Asia (including Korea and Japan). The DIAN core leaders and site investigators, along with representatives from ADAD families and the NIA, formed the DIAN Steering Committee that met annually in conjunction with the ADNI Steering Committee (same day, same venue) because some investigators were involved in both programs (particularly those who were involved with the neuroimaging and neuropathology protocols; Morris had successfully submitted a competing revision in 2007 to the ADNI grant that established the ADNI Neuropathology Core) and also to lay the groundwork for the DIAN‐ADNI comparison study of early onset ADAD (DIAN) and “sporadic” LOAD (ADNI).

When the sample sizes of ADNI and DIAN were sufficiently large to permit comparative analyses, planning began in earnest to examine the hypothesis that ADAD and LOAD share similar longitudinal neuroimaging and fluid biomarker profiles, indicative of a shared pathobiological construct. However, there were two major obstacles to such a comparison. The first obstacle was the marked difference in the age of the DIAN and ADNI cohorts: cognitively normal DIAN mutation carriers had a mean age of 33.5 years (+/−8.8 years) whereas for cognitively normal ADNI participants the mean age was 75.2 years (+/−5.6 years). To address this 4‐decade difference in age, the longitudinal courses for both cohorts were aligned on a clinically meaningful anchor, the age at symptom onset. This alignment adjusted the data across DIAN and ADNI participants for each biomarker and cognitive outcome and allowed longitudinal trajectories to be assessed by estimating and testing the difference in the corresponding slopes between the two cohorts. The second major obstacle was that, over time, the DIAN and ADNI imaging and biomarker protocols were repeatedly modified from those originally proposed and were no longer equivalent for the two studies. To harmonize the data, the magnetic resonance imaging and amyloid PET scans were reprocessed by Dr. Tammie Benzinger of the DIAN Imaging Core at Washington University to guarantee uniform pipelines and ensure equivalency between the cohorts. Furthermore, because DIAN and ADNI used different amyloid radiotracers for the PET studies, the averaged standardized uptake values summarized across multiple brain regions were converted to the Centiloid (CL) scale^8^ to harmonize the tracer and data processing. Pristine aliquots of CSF from DIAN and ADNI participants were processed identically and in singlicate for the biomarker analytes by Dr. Les Shaw of the ADNI Biomarker Core at the University of Pennsylvania. Neither of the NIA grants supporting the DIAN and ADNI studies had budgeted for these reprocessing and harmonization efforts, but the Alzheimer's Association (DIAN_ADNI‐16‐434364) and an anonymous foundation generously funded this work.

This study represented the first longitudinal comparison of identically assessed imaging and CSF biomarkers of AD between ADAD and LOAD. The results indicated that: (1) the longitudinal biomarker profiles for cerebral amyloidosis and tauopathy are similar in both disorders; (2) the rates of cognitive change and hippocampal volume loss in both ADAD and LOAD accelerate after symptom onset; and (3) after symptomatic onset, ADAD has a more aggressive course than LOAD.^9^ These findings suggest a similar pathophysiology for ADAD and LOAD. Although there is no guarantee that results from clinical trials, including prevention trials in preclinical AD, of investigational anti‐Alzheimer drugs in ADAD can be directly extrapolated to LOAD, the results of this comparison study suggest that such extrapolation is possible.

As noted in the Introduction to this Special Issue of *Alzheimer's & Dementia*, ADNI has been a world leader in the standardization and validation of imaging and biofluid markers for AD and has critically supported and improved the growing number of clinical trials of potential disease‐modifying therapies for AD. In doing so, ADNI has also served as the model for other multi‐site longitudinal studies of AD. The formation, conduct, and accomplishments of the DIAN study, which is now led by Dr. Bateman, have benefitted from the pioneering efforts of ADNI, with which it shares the ultimate goal of enabling the success of clinical trials of disease‐modifying therapies for AD.

## CONFLICT OF INTEREST STATEMENT

Neither Dr. Morris nor his family owns stock or has equity interest (outside of mutual funds or other externally directed accounts) in any pharmaceutical or biotechnology company. The authors declare no conflicts of interest. Author disclosures are available in the supporting information.

## Supporting information

Supporting Information
